# Expression, Purification and Low-Resolution Structure of Human Vitamin C Transporter SVCT1 (SLC23A1)

**DOI:** 10.1371/journal.pone.0076427

**Published:** 2013-10-04

**Authors:** Rajendra Boggavarapu, Jean-Marc Jeckelmann, Daniel Harder, Philipp Schneider, Zöhre Ucurum, Matthias Hediger, Dimitrios Fotiadis

**Affiliations:** Institute of Biochemistry and Molecular Medicine, and Swiss National Centre of Competence in Research (NCCR) TransCure, University of Bern, Bern, Switzerland; Swiss Federal Institute of Technology Zurich, Switzerland

## Abstract

Expression and purification of human membrane proteins for structural studies represent a great challenge. This is because micro- to milligram amounts of pure isolated protein are required. To this aim, we successfully expressed the human vitamin C transporter-1 (hSVCT1; SLC23A1) in *Xenopus laevis* oocytes and isolated highly pure protein in microgram amounts. Recombinant hSVCT1 was functional when expressed in oocytes and glycosylated. Structural analysis of purified hSVCT1 by transmission electron microscopy and single particle analysis unveiled its shape, dimensions and low-resolution structure as well as the existence of a major monomeric and minor dimeric population. Chemical crosslinking of isolated oocyte membranes containing expressed hSVCT1 indicated similar oligomeric states of hSVCT1 in lipid bilayers. This work reports the first purification and structural analysis of a human SVCT protein and opens the way for future functional and structural studies using purified hSVCT1.

## Introduction

Vitamin C (ascorbic acid, ascorbate) mediates a variety of enzymatic reactions and acts as an antioxidant against oxidative stress in tissues and cells by scavenging free radicals. In contrast to most higher animals, humans are unable to synthetize L-ascorbic acid, making dietary vitamin C intake essential [1]. This is because of the deficiency of the L-gulono-gamma-lactone oxidase in humans [1], the enzyme catalysing the last step in ascorbic acid biosynthesis from glucose. A well-known human disease linked to deficiency of vitamin C is scurvy. This compromises the synthesis of collagen where vitamin C is required as an electron donor. Other examples for enzymatic reactions involving vitamin C as a cofactor are found in the biosynthesis of carnitine and norepinephrine [2], in adrenal steroidgenesis [3] and in the amidation of peptide hormones [2].

In humans vitamin C is transported across the plasma membrane in a sodium-dependent manner by specialized membrane proteins. These are the sodium-dependent vitamin C transporters SVCT1 (SLC23A1) and SVCT2 (SLC23A2), which are encoded by the SLC23 gene family [4]. This gene family also includes the orphan transporter SVCT3 (SLC23A3) [4]. Phylogenetically, human SVCTs belong to the nucleobase-ascorbate transporter (NAT) family [5], which consists of several hundreds of proteins in all kingdoms of life. Although human SLC23 members belong to the NAT family, no nucleobase transport activity has been reported yet. Besides a distant bacterial NAT homologue [6], no structural information is available on human SVCTs. Hydropathy analysis of human SVCT1 (hSVCT1) suggests that it consists of twelve putative transmembrane domains (TMDs) with intracellular termini [7], [8]. In the extracellular domains of hSVCT1 the asparagine residues Asn138, Asn144 and Asn230 represent potential N-glycosylation sites [7]. Mutagenesis studies of hSVCT1 expressed in COS-1 cells indicated that Asn138 and Asn144 are functional glycosylation sites playing a role in transport activity and targeting to the plasma membrane [8]. The highly conserved proline residues on the extracellular loops between TMD VII and TMD VIII are essential for structural stability and transport activity [9]. SVCT1 co-transports sodium and ascorbate with a 2∶1 stoichiometry [10]. K_m_ values of 65–237 *µ*M were reported [9]. SVCT1 is widely distributed in epithelial tissue of kidney, liver, lung, intestine and skin, where it maintains the whole-body ascorbic acid levels [4].

Variations in the human *SLC23A1* and *SLC23A2* genes were analysed, and *SLC23A2* was shown to be associated with spontaneous preterm delivery [11]. In *Slc23a1* knockout mice death within a few minutes after birth was observed [12] indicating the important role of this transporter in maintaining ascorbate homeostasis in tissues and across the placenta [12], [13].

SVCTs are promising targets for carrier-mediated prodrug approaches. Recently, enhanced oral absorption and systemic bioavailability of a novel prodrug of the anti-HIV protease inhibitor saquinavir was demonstrated when conjugated to vitamin C [14]. Although the protective and therapeutic effects of ascorbate for different cancers are highly debatable, several *in vitro* and *in vivo* studies point into these directions [15]–[17].

Numerous studies are available on the functional roles of SVCT1 and SVCT2, because of their physiological, pharmaceutical and clinical significance. However, no structural studies have been reported so far for SVCT1 and SVCT2. Structural information is important to understand the working mechanisms of these transporters at the molecular level. The primary requirement towards structure determination of human SVCTs, and human membrane proteins in general, is pure, homogeneous and stable protein. The overexpression and purification of eukaryotic and in particular human membrane proteins at levels sufficient for structural studies is often an immense challenge [18]. In the present study, we successfully managed to express functional hSVCT1 in *Xenopus laevis* oocytes and isolate highly pure recombinant protein for structural analysis by negative stain-transmission electron microscopy (TEM) and single particle analysis (SPA). This is the first report describing the purification of a human SVCT, and its low-resolution structure and oligomeric states.

## Materials and Methods

All animal experiments were in accordance with the Swiss Animal Welfare Law and were approved by the Local Veterinary Authority Bern (Veterinäramt Bern; Permit Number: BE 26/12).

### Cloning of hSVCT1 into the pMJB08 Expression Vector

cDNA from hSVCT1 (UniProtKB entry: Q9UHI7) was cloned by PCR from a carrier construct of our laboratory into the pMJB08 expression vector [19]. The forward primer 5′ GGG GAA TTC ATG AGG GCC CAG GAG GAC CTC 3′ and the reverse primer 5′ GGG AAG CTT TCA GAC CTT GGT GCA CAC AGA TGC 3′ were used. PCR products were digested with the restriction enzymes EcoRI and HindIII, and ligated into the pMJB08 vector [19]. The DNA construct was verified by sequencing.

Expression of recombinant hSVCT1 using this construct results in a protein with an N-terminal extension containing and starting with *i.)* a decahistidine tag, *ii.)* a FLAG epitope, *iii.)* a PreScission protease cleavage site and *iv.)* a hemagglutinin (HA) epitope. For more details on pMJB08 see Bergeron *et al.* [19]. The calculated molecular masses of recombinant hSVCT1 based on the amino acid sequence are ∼70.8 kDa (full-length) and ∼67.2 kDa after Prescission protease cleavage.

### Protein Expression in *Xenopus laevis* Oocytes, Isolation of Total Membranes from Oocytes and Protein Purification

Expression of hSVCT1 was performed as previously described for other mammalian and human transporters by Bergeron *et al.* (2011) [19]. This methodological report from our laboratory also describes in great detail the used procedure for the isolation of oocyte membranes containing expressed hSVCT1 and the subsequent affinity purification.

### Determination of Protein Concentration

The bicinchoninic acid (BCA) assay (Pierce, Thermo Scientific) was used for the determination of protein concentrations in isolated oocyte membranes and of purified hSVCT1.

### SDS-PAGE and Immunoblotting of Recombinant hSVCT1

**Figure 1:**
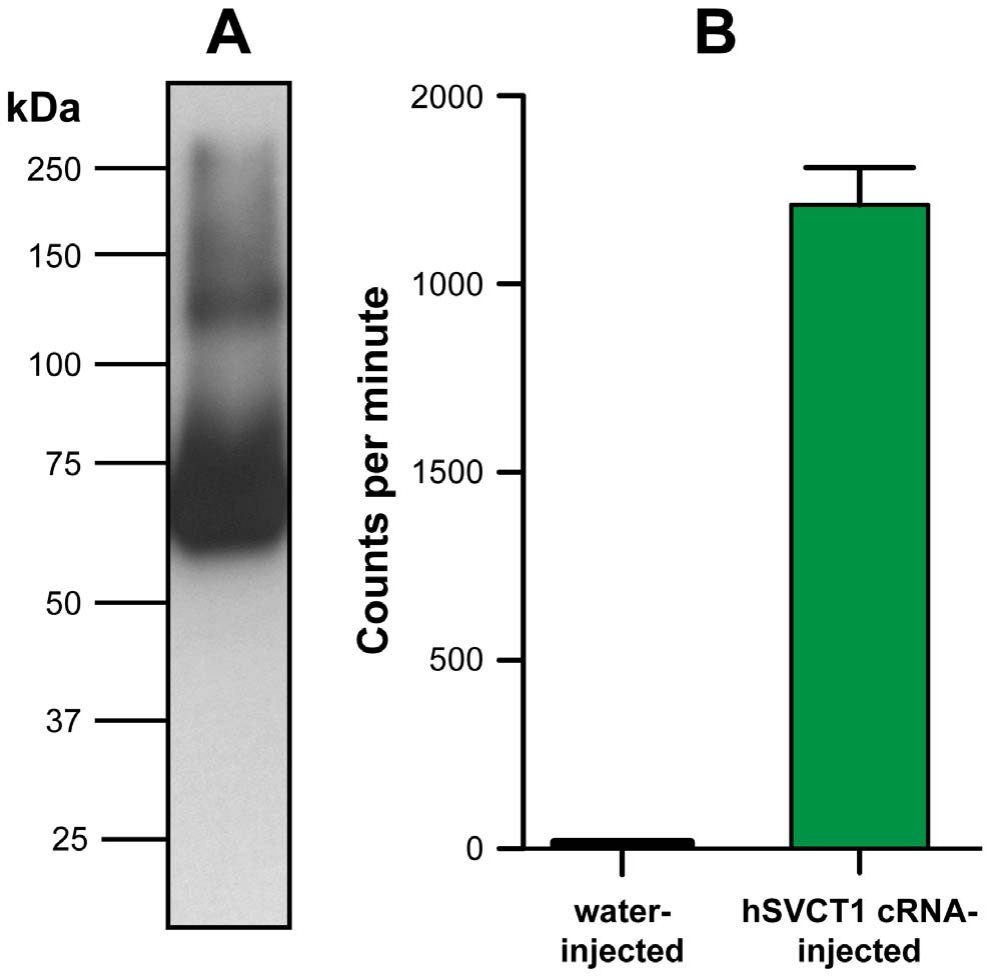
Expression and function of hSVCT1 in *Xenopus* oocytes. (A) Western blot analysis of isolated membranes from three oocytes expressing hSVCT1. Western blotting was performed using a 6% SDS/polyacrylamide gel and an anti-HA antibody. The prominent band below the 75 kDa marker corresponds to recombinant hSVCT1 (calculated molecular mass based on the amino acid sequence: ∼ 71 kDa). A second, less prominent band between the 100 kDa and 150 kDa markers was assigned to dimeric hSVCT1. (B) Oocytes injected with hSVCT1 cRNA mediate uptake of [^14^C]ascorbate in contrast to control oocytes injected with water. Mean ± SEM from 20 oocytes are indicated (two independent experiments).

**Figure 2:**
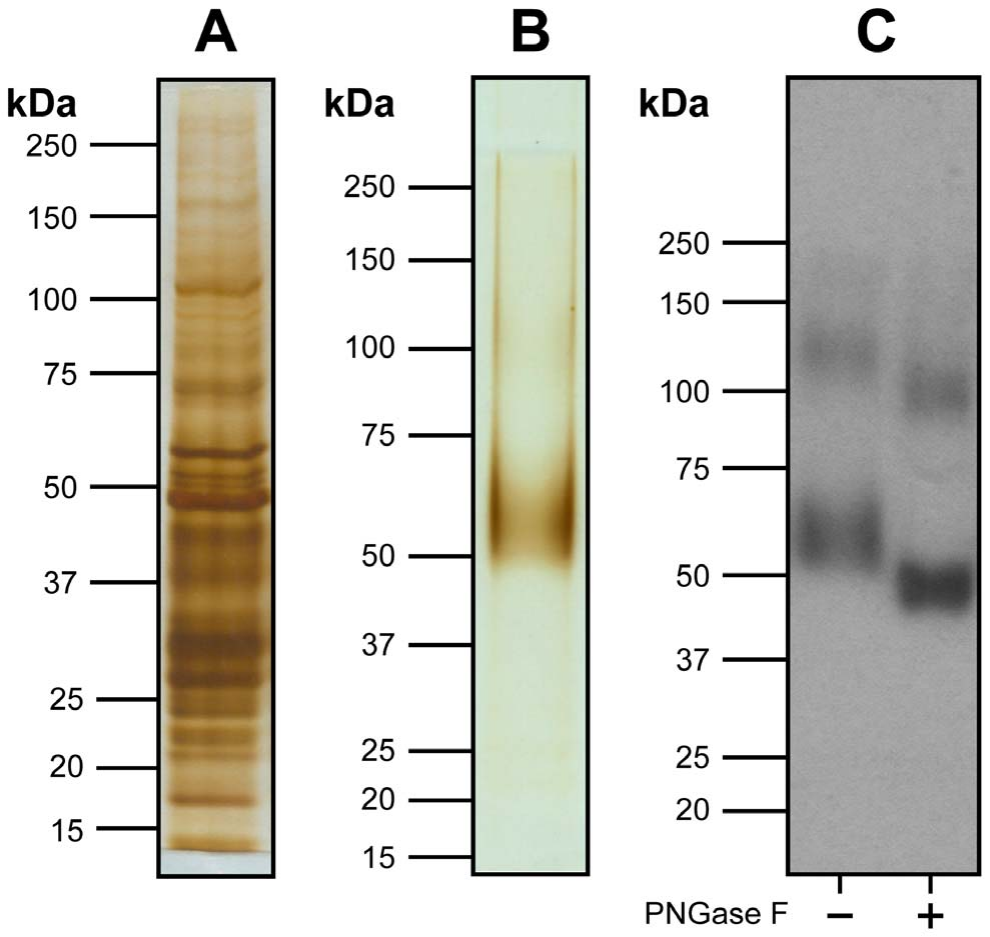
Purification and biochemical characterization of recombinant hSVCT1. Silver-stained SDS/polyacrylamide gels of detergent-solubilized membranes prior binding to cobalt resin (A) and purified hSVCT1 after metal affinity chromatography (B). A prominent hSVCT1 band is discerned between the 50 and 75 kDa markers. (C) Western blot analysis of PNGase F untreated (−) and treated (+) purified hSVCT1. A clear shift towards lower molecular mass and a more compact appearance of the hSVCT1 monomer and dimer bands is observed after PNGase F treatment of purified protein. This indicates *N*-linked glycosylation of hSVCT1 when expressed in *Xenopus laevis* oocytes. In (A)-(C) samples were run on 6% SDS/polyacrylamide gels, and in the Western blot displayed in (C) an anti-HA antibody was used.

SDS-PAGE and Western blot analysis were performed using 6% SDS/polyacrylamide gels (e.g., Fig. 1A and Fig. 2). Gels of isolated oocyte membranes and purified protein were run using Tris-glycine and Tris-Tricine running buffers, respectively. For Western blot analysis PVDF membranes (Immobilon-P Transfer Membrane, Millipore), mouse monoclonal anti-HA antibody (Sigma; primary antibody) and goat anti-mouse IgG (H+L) HRP conjugate (Bio-Rad; secondary antibody) were used.

### Radiotracer Uptake Transport Assay with Xenopus Oocytes

For functional characterization of hSVCT1 expressed in frog oocytes, oocytes were injected with 20 ng of hSVCT1 cRNA. As a control, oocytes were injected with the same volume of water. Oocytes were incubated in Barth’s Medium (MBM: 88 mM NaCl, 1 mM KCl, 2.4 mM NaHCO_3_, 0.82 mM MgSO_4_, 0.66 mM NaNO_3_, 0.75 mM CaCl_2_, 10 mM HEPES-NaOH (pH 7.4)) and supplemented with P/S antibiotics (GIBCO™ Penicillin-Streptomycin liquid, Invitrogen) for three days at 18°C. On the fourth day, 10 oocytes were transferred into a centrifuge tube with 200 *µ*l of MBM containing 0.5 *µ*Ci [^14^C]ascorbate at 450 *µ*M final concentration (specific activity 0.00535 Ci/mmol, Perkin Elmer). After 15 min incubation at room temperature, the radioactive liquid was removed from the tube, and the oocytes were washed three times with 1 ml of cold MBM buffer. Oocytes were then transferred individually into the wells of a 96 well plate and 50 *µ*l of 5% SDS solution was added. The wells with oocytes were sealed and put on a shaker operated at 900 rpm for 25 min to lyse and homogenize the oocytes. 150 *µ*l of scintillation cocktail (Microscint-40, Perkin Elmer) were added to each well. Finally, wells were resealed and uptake counts were measured using a Packard TopCount scintillation counter.

### Deglycosylation of Purified hSVCT1

For deglycosylation, 40 *µ*l (2.4 *µ*g) of purified hSVCT1 in 20 mM Tris-HCl (pH 8), 150 mM NaCl, 0.05% Triton X-100 were incubated with 8 *µ*l PNGase F (New England Biolabs; final PNGase F concentration of 0.046 *µ*g/*µ*l) for 30 min at room temperature. Similarly and as negative control, purified hSVCT1 was incubated in absence of PNGase F. For SDS-PAGE and Western blot analysis, equal volumes of 5×SDS sample buffer were added to the two reaction mixtures. For Figure 2C, ∼250 ng of untreated and PNGase F-treated hSVCT1 were loaded on a 6% SDS/polyacrylamide gel, and subjected to SDS-PAGE and immunoblotting.

### Negative-stain TEM and SPA

Purified hSVCT protein at ∼60 *µ*g/ml was adsorbed for ∼30 seconds to parlodion carbon-coated copper grids which were rendered hydrophilic by glow discharge at low pressure in air. Grids were washed with three drops of double-distilled water and stained with two drops of 0.75% uranyl formate. Electron micrographs were recorded at a magnification of x110,000 with a Philips CM12 TEM operated at 80 kV and equipped with a Morada 11 megapixel CCD camera (Soft Imaging System).

**Figure 3:**
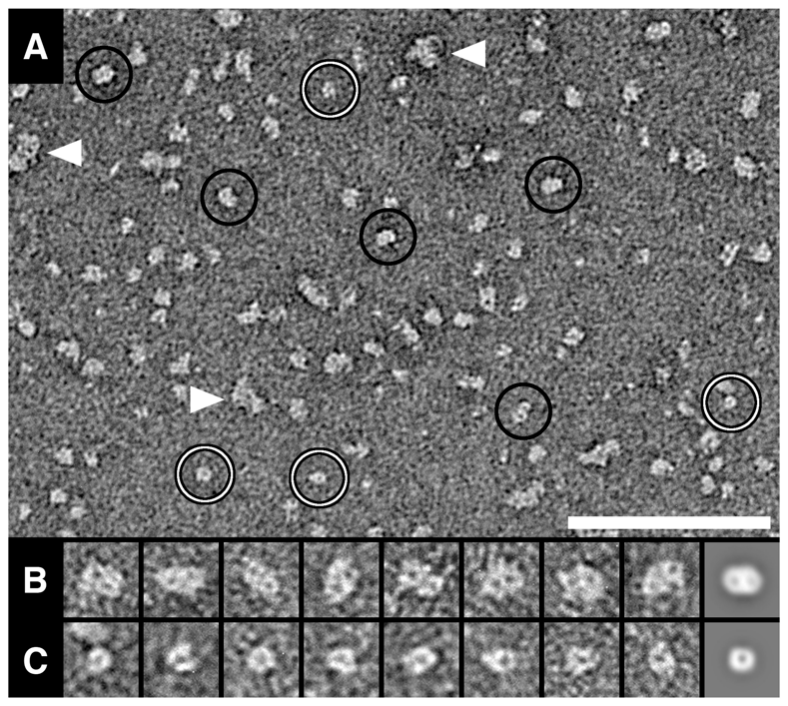
Negative stain-TEM and SPA of purified hSVCT1. (A) Overview electron micrograph of purified hSVCT1 adsorbed on a parlodion carbon-coated surface. Projections of differently oriented hSVCT1 proteins are discerned. Large, elliptical (hSVCT1 dimers) and small, round-shaped (hSVCT1 monomers) particles are marked by black and white circles, respectively. Small protein aggregates are also observed (arrowheads). Well-preserved top views of dimers (B) and monomers (C) were magnified and are displayed in the gallery. The rightmost frames in (B) and (C) display averages of dimer (n = 387) and monomer (n = 316) top views, respectively. In hSVCT1 dimers (B) two stain accumulations are discerned, while in monomers (C) only one. Averages are low-pass filtered to 2 nm resolution. The scale bar in (A) is 100 nm. The frame sizes in (B) and (C) are 18.5 nm.

Single particle analysis and averaging of negatively stained, purified hSVCT1 proteins were performed using the EMAN software package [20]. The first Thon ring on power spectra of CCD images selected for SPA was typically between (1.0 nm)^−1^–(1.8 nm)^−1^. The best preserved 366 monomer top views and 455 dimer top views of hSVCT1 were picked from CCD images. Individual reference free class averages of monomers and dimers were calculated and used to compute the presented averages (originating from 316 monomer and 387 dimer particles). Averages were low-pass filtered to 2 nm resolution. No two-fold symmetry was imposed to the dimer average (Fig. 3B, rightmost frame).

### Chemical Crosslinking of hSVCT1 in Isolated Oocyte Membranes

**Figure 4:**
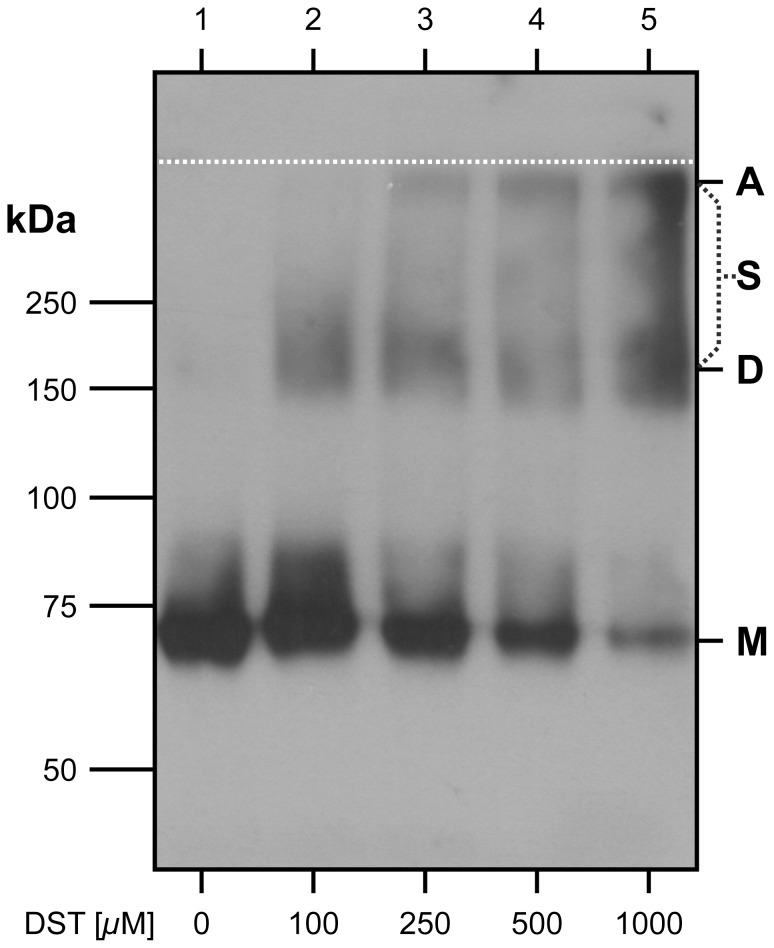
Chemical crosslinking of hSVCT1 in isolated oocyte membranes. Western blot analysis of isolated oocyte membranes containing hSVCT1 incubated at different DST concentrations, i.e., 0, 100, 250, 500 and 1000 *µ*M. hSVCT1 monomer (**M**), dimer (**D**) and aggregate (**A**) bands are indicated. At ≥250 *µ*M DST increasing smearing (**S**) and aggregation, and decreasing amounts of monomers are observed indicating unspecific crosslinking. Western blotting was performed using a 6% SDS/polyacrylamide gel and an anti-HA antibody. The interface between stacking and separating gels is indicated (dotted, white line).

Chemical crosslinking of hSVCT1 in isolated oocyte membranes was performed with the homobifunctional amine-reactive crosslinker disuccinimidyl tartrate (DST; spacer arm length: 6.4 Å; Pierce, Thermo Scientific). For crosslinking, membranes from 1,000 oocytes were isolated as previously described [19], but using 20 mM HEPES-NaOH (pH 7.5) instead of 20 mM Tris-HCl (pH 8) in all buffers. This is necessary because Tris contains a primary amine that competes with amine-reactive crosslinkers such as DST. Total protein concentration of oocyte membranes was adjusted to 1 mg/ml. Chemical crosslinking with DST was performed at four different concentrations: 100 *µ*M, 250 *µ*M, 500 *µ*M and 1000 *µ*M of DST for 15 min at 15°C, and at a total protein concentration of 100 *µ*g/ml. The crosslinking reaction was terminated by adding glycine to a final concentration of 50 mM. Equal volumes of 5×SDS sample buffer were then added to the reaction mixtures. In Figure 4, ∼450 ng of total protein were loaded on a 6% SDS/polyacrylamide gel and subjected to SDS-PAGE and immunoblotting.

## Results and Discussion

Frog oocytes have been shown to be ideal for expression and purification of human membrane transporters [19]. Purified proteins of different recombinant transporters were of high quality and the isolated amounts suitable for structural analysis by transmission electron microscopy (TEM) and single particle analysis (SPA) [19]. Compared to most expression systems, which work in cell culture, the effort is higher because single oocytes have to be injected with cRNA. However, advances in automatization have been done during the last years, making the cRNA-microinjection of a few thousand oocytes per day possible. Here, automated injection of Xenopus oocytes was applied for expression of hSVCT1.

### Expression and Function of hSVCT1 in Xenopus Oocytes

The cDNA of hSVCT1 was cloned into the oocyte expression vector pMJB08 [19]. Expression results in a recombinant protein with decahistidine tag, and FLAG and HA epitopes at the N-terminus. In addition, a PreScission protease cleavage site is present between the FLAG and HA epitopes. Following the method by Bergeron *et al.* [19], cRNA was injected into frog oocytes (20 ng per oocyte) and protein expression tested after three days of incubation at 18°C. Figure 1A displays a representative Western blot of isolated total membranes from 3 oocytes with a prominent band visible between the 50 and 75 kDa markers. This band corresponds to recombinant hSVCT1, which has a calculated molecular mass of ∼71 kDa based on the amino acid sequence. A second weaker band was visible between the 100 and 150 kDa markers, thus about twice the molecular mass of the previous one and corresponding to hSVCT1 dimers. In a next step, uptake experiments using water-injected and hSVCT1 cRNA-injected oocytes, and radiolabeled vitamin C were performed. As shown in Figure 1B, cRNA-injected oocytes clearly indicated uptake of [^14^C]vitamin C and thus functionality of recombinant hSVCT1.

### Purification and Biochemical Characterization of Recombinant hSVCT1

For protein purification, total membranes from 1,200–5,000 oocytes expressing hSVCT1 were isolated and solubilized with the non-ionic, mild detergent Triton X-100. After ultracentrifugation, the supernatant (Fig. 2A) containing detergent-solubilized, His-tagged hSVCT1 was subjected to cobalt affinity chromatography. hSVCT1 was then specifically detached from the resin by on-column cleavage with the protease PreScission, i.e., by removal of the N-terminal end containing the His-tag. Because this protease is His-tagged and binds to the cobalt resin, cleaved hSVCT1 could be eluted from the chromatography column without contamination from PreScission. Protein yields from five independent hSVCT1 expressions/purifications were 6, 6, 16, 22 and 48 *µ*g from 1,200, 1,500, 5,000, 3,500 and 5,000 injected oocytes, respectively. From these numbers an average expression of ∼6 ng of hSVCT1 per oocyte was estimated. Figure 2B displays a typical silver-stained SDS/polyacrylamide gel of purified hSVCT1 documenting the high purity of the isolated protein. Similar to Figure 1A, hSVCT1 migrated between the 50 and 75 kDa markers. A hSVCT1 dimer band was not obvious in the silver-stained gel (Fig. 2B) due to the low abundance of the dimer and the detection limit of silver staining. However, a hSVCT1 dimer band was observed on Western blots of purified hSVCT1 (Fig. 2C, -) indicating the presence of a minor population of hSVCT1 dimers. Importantly, there were no indications for protein degradation or contamination (Fig. 2B). Diffuse bands were characteristic of hSVCT1 on SDS-PAGE gels. To test, if the diffuse bands arose from *N*-linked glycosylation of the protein, purified hSVCT1 protein was incubated with the endoglycosidase PNGase F and subjected to Western blot analysis (Fig. 2C). Compared to untreated (Fig. 2C, -), PNGase F-treated (Fig. 2C, +) hSVCT1 clearly indicated a change in migration towards lower molecular mass and a more compact appearance of hSVCT1 bands on Western blots. This supports the presence of *N*-linked glycosylation in hSVCT1 expressed in frog oocytes.

### TEM and SPA of Purified Recombinant hSVCT1

For structural analysis, purified hSVCT1 was negatively stained and examined in the TEM. Figure 3A displays a typical electron micrograph of hSVCT1 protein adsorbed on a carbon-coated parlodion grid. Because proteins can adsorb in different orientations on a support, their projections on electron micrographs reflect different views of the protein, e.g., top and side views. Two distinct top view populations of particles were found: larger, elliptical particles with two stain accumulations (Fig. 3A, black circles) and smaller, round-shaped particles with a central stain accumulation (Fig. 3A, white circles). Small protein aggregates were also observed (Fig. 3A, arrowheads). The galleries in Figure 3 display selections of well-preserved top views of larger (B) and smaller (C) hSVCT1 particles. The rightmost frames in these two galleries are calculated averages of the corresponding particle populations. From these averages, dimensions of ∼8.2 nm x ∼11.1 nm were estimated for the larger, elliptical particles and a diameter of ∼7.7 nm for the smaller, round-shaped particles. Dimensions, shapes and low-resolution structures of the two distinct particle populations suggest the presence of monomeric and dimeric hSVCT1 when purified in detergent. Estimated from particles on electron micrographs (Fig. 3A), dimers were minor compared to monomers.

### Chemical Crosslinking of hSVCT1 in Isolated Oocyte Membranes

To explore if dimeric hSVCT1 proteins also exist in membranes, chemical crosslinking experiments of isolated oocyte membranes expressing hSVCT1 with the homobifunctional crosslinker disuccinimidyl tartarate (DST; spacer arm 6.4 Å) were performed. Figure 4 displays a Western blot of isolated oocyte membranes containing hSVCT1 incubated at different concentrations of DST. In contrast to membranes incubated in the absence of DST (Fig. 4, lane 1), the presence of crosslinker induced hSVCT1 dimer formation (Fig. 4, lanes 2–5; dimer band: **D**). Although not clearly visible in Fig. 4, lane 1 a minor population of SDS-resistant hSVCT1 dimers (relatively to monomers) is present on SDS-PAGE gels of non-crosslinked membranes (see Fig. 1A for a longer developed blot). Optimal conditions were at 100 *µ*M DST, where basically only hSVCT1 monomers and dimers were detected (Fig. 4, lane 2). At 250, 500 and 1000 *µ*M (Fig. 4, lanes 3–5) increasing smearing (**S**) and amounts of aggregation (band: **A**), and decreasing amounts of monomer (band: **M**) were observed. Smearing and aggregation below the stacking and separating gel interface (Fig. 4; dotted, white line) are characteristic of crosslinking due to random collision, i.e, unspecific crosslinking between hSVCT1s and other proteins of varying molecular masses. In summary, crosslinking indicated the presence of hSVCT1 dimers in isolated membranes, although at significant lower amounts compared to monomeric hSVCT1 (Fig. 4, lane 2).

## Conclusion

Expression, purification and structure determination of human membrane proteins remains a great challenge. Isolation of microgram amounts of recombinant protein made possible the determination of shapes, dimensions, low-resolution structures and oligomeric states of hSVCT1. To our knowledge, this is the first report on the successful purification of a SLC23 gene family member and paves the path for future functional and structural studies using purified hSVCT1, e.g., functional reconstitution and crystallization.
